# Nutrient-extraction blender preparation reduces postprandial glucose responses from fruit juice consumption

**DOI:** 10.1038/nutd.2017.36

**Published:** 2017-10-09

**Authors:** K M Redfern, V L Cammack, N Sweet, L A Preston, SoBHCS Student Team, M A Jarvis, G A Rees

**Affiliations:** 1 School of Biomedical and Healthcare Sciences, Plymouth University Peninsula Schools of Medicine and Dentistry, Plymouth, UK

## Abstract

Although whole-fruit consumption is regarded as protective against type 2 diabetes (T2DM), conventionally prepared fruit juice is associated with increased T2DM risk, and current public health advice recommends its restriction. ‘Nutrient extractor’ style blenders are increasing in popularity worldwide as an alternative means of juicing fruit, but little is known about their effect on postprandial glucose levels. The current study investigated the effect of nutrient extraction on postprandial blood glucose response and glycemic index (GI) compared with a glucose control for both mixed fruit and a high GI fruit (mango). Remarkably, consumption of nutrient-extracted mixed fruit resulted in a significant lowering of the GI (32.7±8.5) compared with whole mixed fruit (66.2±8.2, *P*<0.05). For the high GI mango, there were no differences between nutrient-extracted and whole fruit, indicating that even for a high GI fruit the effect of nutrient extraction does not increase GI compared with the whole fruit. These findings suggest that, in contrast to conventionally prepared fruit juice, fruit juice prepared by nutrient extraction in some cases elicits a more favorable postprandial glycemic response than whole fruit and even for high GI fruits do not worsen the response. The mechanism responsible for this effect is currently unclear. However, these results suggest that fruit homogenized by nutrient extraction should be considered as a potential dietetic strategy for glycemic control.

## Introduction

Increased fruit consumption is associated with the lowering of risk from multiple chronic diseases;^1^ and public health campaigns worldwide consistently aim to promote awareness of the positive health effects of fruit. However, current UK public health guidance, including from Diabetes UK and the NHS, recommends limiting consumption of fruit juice to 150 ml per day. The rationale behind this advice is, in large part, based on a >70 000 female nurse cohort study from the United States^2^ which when pooled with two other studies showed an association of fruit juice consumption with increased risk of type 2 diabetes (T2DM).^3^ Consumption of whole fruit was not associated with T2DM risk but was actually associated with lowered risk. The authors proposed that the increase in glycemic load and decrease in fiber per serving of fruit juice compared with whole fruit may explain the increased risk of T2DM. However, fruit restriction in a group of newly diagnosed type 2 diabetics had no impact on glycated hemoglobin, although both the intervention and control groups were advised to exclude fruit juice entirely from their diets.^4^ Furthermore, two recent meta-analyses were not able to identify enough conclusive evidence to support an association between fruit juice consumption and T2DM.^5, 6^

The increasing popularity of ‘nutrient-extractor’ style blenders in the United Kingdom suggests that the general public, as well as patients already exhibiting risk factors for T2DM, are consuming fruit in a new way for which the health risks remain unclear. Unlike traditional juicers that remove the pulp leaving only the juice, these blenders homogenize the whole fruit without removing fiber. Given the popularity of nutrient extractors for fruit preparation, it is critical to understand the impact of this method of juicing fruit on postprandial glycemic response in order to provide up to date public health guidance. The present study compared the postprandial glycemic response of fruit homogenized using a commercially available nutrient extractor against whole fruit and a glucose control in healthy volunteers.

## Subjects and methods

Participants were healthy student volunteers (mixed fruit arm, males *n*=7, females *n*=12, age 20–24 years; mango arm, males *n*=4, females *n*=5, age 20–27 years). Sample sizes were based on methods described by Brouns and colleagues.^7^ All participants answered a health questionnaire, which included questions regarding their present health status, medication use and medical history. Exclusion criteria were body mass index >30 kg m^−2^, pregnancy, fruit allergy, known diabetes, glucose intolerance or insulin resistance, use of medication known to interfere with glucose homeostasis or intestinal absorption. Written informed consent was obtained from each participant, and the study was approved by the Research Ethics Committee of the Faculty of Science and Engineering at the University of Plymouth.

### Materials/processing

For each arm of the study, the two test meals: (i) whole fruit or (ii) nutrient-extracted fruit both contained 25 g total sugar per serving.^8^ For the mixed fruit arm, this consisted of the following: banana (25 g), mango (25 g), passion fruit (50 g), pineapple (50 g), kiwi (50 g) and raspberries (50 g), while for the mango arm it was mango alone (181 g). All fruits were washed, peeled and cut into bite-sized pieces. Nutrient-extracted servings were processed in a 600 W, 20 000 r.p.m. blender (Nutribullet 600, Nutribullet LLC, Pacoima, CA, USA) for 30 s with 125 ml water, transferred to a plastic cup, sealed and frozen. Whole-fruit servings were transferred to a container and frozen. Test meals were transferred to a refrigerator the day before test days and allowed to defrost overnight. Control meals for both arms were prepared on the morning of testing and consisted of 25 g glucose dissolved in 125 ml water.

### Experimental procedure

A crossover design was used for this study, so each participant served as their own control. Each participant consumed each test meal, with a minimum 3-day washout period between test days. Participants were required to fast for 12 h and to avoid alcohol, caffeine and vigorous exercise in the 24 h preceding testing.

Participants arrived at the University of Plymouth’s Nutrition, Exercise and Health Laboratories on each testing day at 0900 hours. Fasting glucose levels were obtained via a finger prick blood sample (Accu-Check Advantage, Roche, Welwyn Garden City, UK), which has compared favorably with laboratory testing in previous literature.^9, 10^ Testing was considered to begin at first oral contact with the test meal, which was consumed steadily over a 15-min period, alongside 125 ml of water for the whole-fruit group. Postprandial blood glucose levels were determined at 15, 30, 45, 60, 75, 90, 105 and 120 min for each test meal, with each participant using the same glucometer throughout the study, which was calibrated prior to study commencement according to the manufacturer’s instructions.

The glycemic index (GI) was calculated from the incremental area under the 2-h glucose response curve for each test meal:^11^


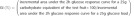


The incremental area under the curve for each test meal was expressed as the percentage of the mean area under the control curve for the same subject. These values were used to calculate GI values for each test meal using methods described by Brouns *et al.*^7^

### Statistical analysis

All statistical analyses were performed using the SPSS 23 Statistical Software (IBM Corp, Armonk, NY, USA). A one-way repeated-measures analysis of variance was conducted to determine whether significant differences existed in GI between test meals. Mauchly’s test of sphericity was non-significant, which indicates that the assumption of sphericity was met. All data were presented as means±s.e. unless otherwise stated, with the significance set at *P*⩽0.05.

## Results

### Mixed fruit arm

**Figure 1:**
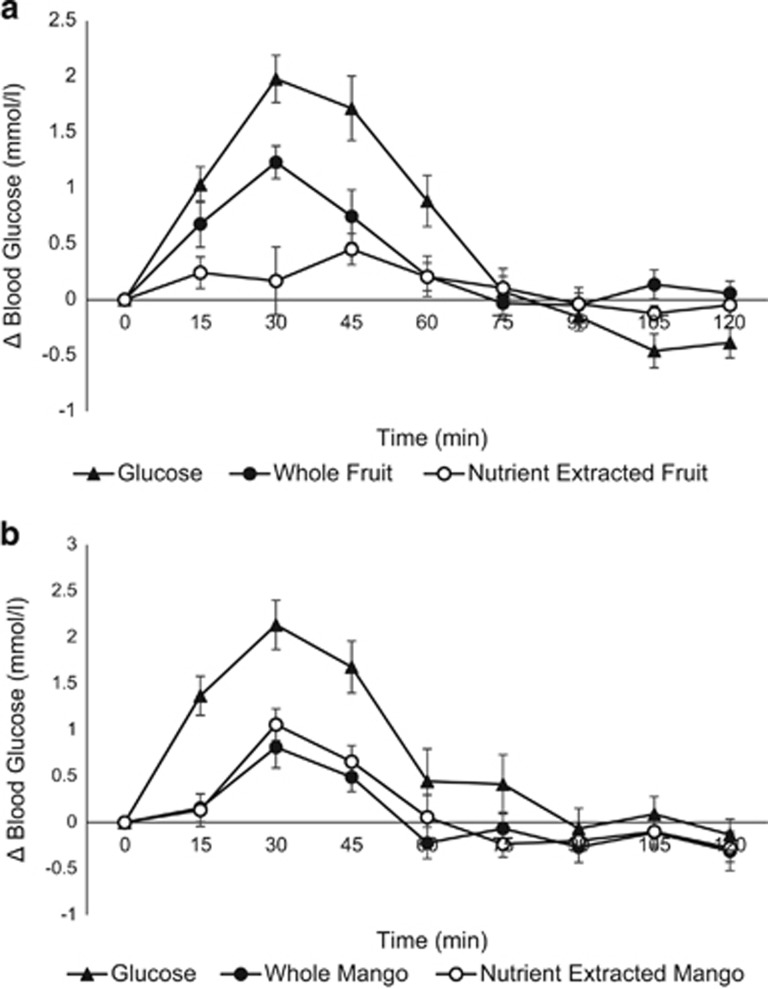
Comparison of the incremental area under the curve values of blood glucose levels after ingestion of the glucose control, whole fruit and nutrient-extracted fruit. (**a**) Mixed fruit; (**b**) Mango alone.

The postprandial glucose responses after consumption of whole fruit, nutrient-extracted fruit and glucose (control) are shown in Figure 1a. The glucose meal elicited the greatest rise above preprandial levels and declined below preprandial levels, while whole fruit and nutrient-extracted fruit returned to preprandial levels. Glucose and whole fruit peaked at 30 min, while nutrient-extracted fruit peaked later at 45 min, with a slower rise and decline in blood glucose.

**Figure 2:**
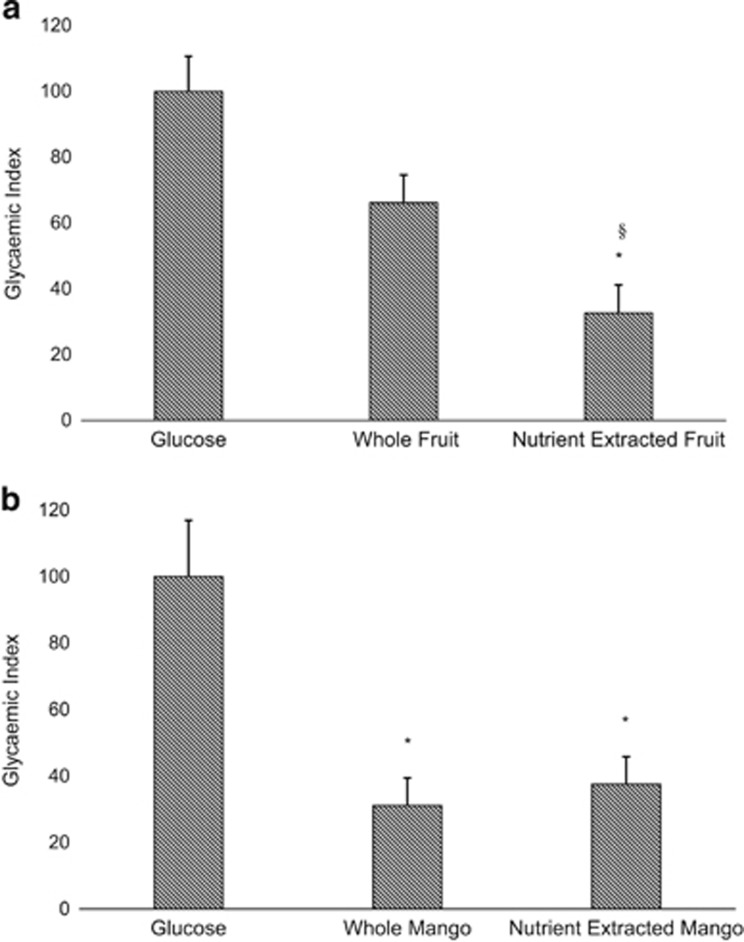
Comparison of GI for each test meal. (**a**) Mixed fruit; (**b**) Mango alone *P<0.05 compared with glucose control, §*P*<0.05 compared with the whole fruit.

Figure 2a shows the GI of the three test meals as calculated from the incremental area under the curve. The mean GI for nutrient-extracted fruit was significantly lower (32.7±8.5) than whole fruit (66.2±8.2, *P*<0.05) and glucose control (100.0±10.7, *P*<0.05).

### Mango arm

The mean GI for whole mango (31.1±8.2) was not significantly different to that of nutrient-extracted mango (37.6±8.2), *P*=1.00). Mean GI for both whole and nutrient-extracted mango were significantly lower than glucose (100±16.9, *P*<0.05) (Figure 2b).

## Discussion

Results from our study indicated that nutrient extraction of fruit has the potential to significantly lower its GI. Notably, the effect of nutrient extraction resulted in a lowered GI response even than consumption of the corresponding whole fruit, with whole fruit being associated with a medium GI between 55 and 69, whereas the nutrient-extracted fruit juice had a low GI ⩽55.^11^ Although the mango arm of our study did not follow the same pattern, nutrient-extracted preparation of the mango juice did not adversely influence GI compared with whole fruit, with both preparations exhibiting a low GI. These finding may have important clinical implications for healthy people, as well as T2DM patients, who are currently following recommendations to avoid fruit juice in the hope of improving glycemic control. Low GI diets have been shown to improve body weight, glycemic control and glycated hemoglobin levels.^12, 13^ Improvements in these risk factors can reduce the risk of developing T2DM and reduce the need for medication, improve quality of life and reduce the risk of complications for those already living with T2DM.

Earlier findings of Elizondo-Montemayor *et al.*^14^ using high hydrostatic pressure showed that processing of food can alter GI responses. Other studies have shown low GI fruit, but not fruit juice, to positively affect glycemic control^3, 4^ and reduce the risk of developing T2DM. However, our current study is, to our knowledge, the first to assess the effect of nutrient-extracted fruit on GI responses. The rationale behind current public health advice to limit the consumption of fruit juice to 150 ml per day is based on the theory that juicing releases intrinsic sugars from cells and removes insoluble fiber, which in turn increase postprandial glucose response.^15^ In the case of nutrient-extractor-based homogenization, none of the fruit is removed thus both insoluble and soluble fiber remain in solution. There are a number of possible models by which nutrient extraction alters GI. For example, the presence of amylase in saliva may accelerate the hydrolysis of starch during and after mastication for the whole-fruit group thus increasing the rate at which glucose can be absorbed compared with the nutrient-extracted fruit. The precise mechanism by which the nutrient extraction attenuates the postprandial response remains unclear and is an area of ongoing research in the laboratory.

Glucometers were chosen as a convenient means to measure glycemic response to test meals in the current study. The use of glucometers is one potential limitation of the study as this method of analysis has been shown in some cases to provide an overestimate of blood glucose levels compared with other methods.^16, 17^ However, when correctly calibrated as in the present study, the level of overestimation can be regarded as a constant and thereby viewed as a reliable means of monitoring individual variation in blood glucose that is comparable with values obtained by perhaps more accurate methodologies.^9, 10^ Use of medication known to affect glucose metabolism was an exclusion criterion; however, female participants were not asked about their use of oral contraceptives, which we acknowledge may have influenced glycemic response across the test period. However, the pattern of glycemic response was similar between male and female participants, and GI was not significantly different between males and females for each test meal (data not shown).

In summary, our present study demonstrates the potential of commercially available nutrient-extractor-type blenders to reduce GI associated with consumption of fruit juice. Ongoing and future work is examining the effects of nutrient extraction on postprandial responses of individual fruits and fruit mixtures alongside nutritional analysis. However, this initial study identifies nutrient extraction as one means by which fruit juice can be consumed within a dietetic strategy for glycemic control.
